# The Thalamus as a Blackboard for Perception and Planning

**DOI:** 10.3389/fnbeh.2021.633872

**Published:** 2021-03-01

**Authors:** Robert Worden, Max S. Bennett, Victorita Neacsu

**Affiliations:** ^1^Wellcome Centre for Human Neuroimaging, Institute of Neurology, University College London, London, United Kingdom; ^2^Independent Researcher, New York, NY, United States; ^3^Bluecore, New York, NY, United States

**Keywords:** thalamus, blackboard architecture, Bayesian cognition, spatial steering, supervised learning, pulvinar, MD nucleus, paraventricular nucleus

## Abstract

It has been suggested that the thalamus acts as a blackboard, on which the computations of different cortical modules are composed, coordinated, and integrated. This article asks what blackboard role the thalamus might play, and whether that role is consistent with the neuroanatomy of the thalamus. It does so in a context of Bayesian belief updating, expressed as a Free Energy Principle. We suggest that the thalamus-as-a-blackboard offers important questions for research in spatial cognition. Several prominent features of the thalamus—including its lack of olfactory relay function, its lack of internal excitatory connections, its regular and conserved shape, its inhibitory interneurons, triadic synapses, and diffuse cortical connectivity—are consistent with a blackboard role.Different thalamic nuclei may play different blackboard roles: (1) the Pulvinar, through its reciprocal connections to posterior cortical regions, coordinates perceptual inference about “what is where” from multi-sense-data. (2) The Mediodorsal (MD) nucleus, through its connections to the prefrontal cortex, and the other thalamic nuclei linked to the motor cortex, uses the same generative model for planning and learning novel spatial movements. (3) The paraventricular nucleus may compute risk-reward trade-offs. We also propose that as any new movement is practiced a few times, cortico-thalamocortical (CTC) links entrain the corresponding cortico-cortical links, through a process akin to supervised learning. Subsequently, the movement becomes a fast unconscious habit, not requiring the MD nucleus or other thalamic nuclei, and bypassing the thalamic bottleneck.

## Introduction

The thalamus occupies a central position in the brain. Because of its volume and extensive cortical connections (Sherman and Guillery, 2006) it has significant metabolic costs. Compared to its cost and central position, many theories of thalamic function have an unsatisfactory aspect; if the thalamus is merely a relay for sense data, or merely an enforcer of cortical rhythms, or a controller of arousal, why devote all that expensive brain real estate to such simple functions, which could perhaps be done more locally in cortex or brainstem? These thalamic functions do not seem to license the significant cost of the thalamus.

These are not the only theories of thalamic function. It has been proposed (Baars, 1988; Mumford, 1991; O’Reilly et al., 2017, 2020; Dehghani and Wimmer, 2018) that the thalamus acts as a blackboard or global workspace in the brain. Blackboard functions appear sufficiently important to justify having the thalamus; but what exactly do they mean? What does a blackboard do? Is the neuroanatomy and neurophysiology of the thalamus consistent with a blackboard function?

This article addresses those questions, relating the blackboard proposals to Bayesian inference in the brain, as entailed by the Free Energy Principle (Friston, 2003). In this article, we will use spatial cognition, planning of movements, and risk-reward trade-off decisions as three examples to illustrate the role of the thalamus as a central blackboard, central in terms of its physical location and role in information processing. This will be evinced through its highly specific anatomical structure and underlying connectivity as a blackboard system. In this role, the thalamus can be regarded as instantiating a generative model of objects in a three-dimensional peripersonal space.

This article explores two key ideas:

• **The thalamus is a blackboard for cognition**, particularly for 3-D spatial cognition, movement planning; and risk-reward trade-offs.
• **The thalamus entrains the cortex**, so that routine movement tasks and possibly also risk-reward decisions are taken over by cortico-cortical links, releasing thalamic resources for more context-sensitive processing.

In a formal approach to planning and decision making, planning can be realized through probabilistic inference, expressed as minimization of free energy (Attias, 2003; Baker et al., 2009; Botvinick and Toussaint, 2012; Mirza et al., 2016; Kaplan and Friston, 2018). In this formulation, the agent builds a (minimum free energy) internal model of the world (including its 3D spatial structure). This is usually referred to as a generative model. Then the agent represents its beliefs about the future, present, and the past as joint probability distributions over states, actions, and consequent outcomes in the future (Kaplan and Friston, 2018).

The other side of the coin, learning to infer, habituates this implicit inference enabling fast and automatic recognition of the most likely cause of sensations—and the aptest behavioral responses (Friston, 2003). In Machine Learning, this process has also been referred to as “amortization” (Gershman and Goodman, 2014). In what follows, we explore the potential roles played by the thalamus within these frameworks, specifically through its modulatory role in the attentional selection and subsequent “voting” in cortico-cortical entrainment.

These proposals articulate a central and vital role for the thalamus and agree with many experimental findings. Spatial cognition and the control of movement are cardinal functions of animal brains; but spatial cognition is hard, involving precise geometry and the fusion of multi-modal sense data, which require a scarce central resource—the thalamus—also implying a need to delegate routine tasks to cortex quickly and efficiently, by configuring cortico-cortical pathways.

## Theories of Thalamic Function

For many years, the prevailing characterization of the function of the thalamus has been that it acts as a relay. This idea was derived originally from consideration of primary thalamic nuclei such as the LGN, which evidently relay information from sense organs to the cortex (Jones, 2007). The idea has been extended to higher-order thalamic nuclei such as the Pulvinar and the MD nucleus—suggesting that those nuclei relay information between cortical regions and subcortical regions.

In recent years, evidence from thalamic neurophysiology and its engagement by diverse cognitive tasks have brought the “relay” interpretation into question (Sherman, 2007), especially for higher-order thalamic nuclei. There are two main reasons for this. First, studies of thalamocortical neuroanatomy speak of an increasingly complex picture (e.g., Halassa and Sherman, 2019), of the thalamus connected to diverse cortical regions, which have a wide range of different functions, *via* different types of circuit “motif” including driving and modulatory connections, both focal and diffuse, convergent and divergent (Shipp, 2003; Sherman and Guillery, 2006; Barron et al., 2015; Homman-Ludiye et al., 2020). The most remarkable aspect of thalamic neuroanatomy—that the thalamic nuclei are close yet have no lateral connections to one another (Sherman and Guillery, 2006)—remains unexplained. Similarly, the unique anatomy of the TRN (Pinault and Deschênes, 1998) has yet to be accounted for.

Second, studies suggest thalamic involvement in a very wide range of cognitive functions, including perception, attention (Saalman and Kastner, 2014; Wimmer et al., 2015; Schmitt et al., 2017), memory (Dumont and Aggleton, 2013; Warburton, 2018), task engagement (Marton et al., 2018), learning, motor control (Ouhaz et al., 2018), and executive decision-making (Do Monte et al., 2015). Put simply, the accumulating evidence about the role, importance, and connectivity of the thalamus has outrun the “thalamus as a relay” picture. The notion that cognitive processing is restricted to the cortex—with the thalamus acting as a kind of message boy—appears to be untenable. To quote Sherman (2017): “The conventional, textbook view of thalamocortical interactions needs a drastic makeover.” In short, higher-order thalamic nuclei may not merely pass information among cortical regions. We need to ask what those nuclei do to—or with—this information, as it traverses the thalamocortical loops.

In the light of the above, it seems unlikely that there will ever be an exclusive theory of thalamic function, that the thalamus does “this and only this.” Several proposals are now gaining traction that may not be mutually exclusive. Some examples include: passing efference copies of motor controls to cortical regions, to help to anticipate their effects (Sherman, 2016); controlling information/cost trade-offs (Dehghani and Wimmer, 2018); updating mental representations (Wolff and Vann, 2019); playing a role in solving the binding problem (Treisman, 1998), entraining oscillations between disparate cortical areas to coordinate processing (Malekmohammadi et al., 2015); deep predictive learning (O’Reilly et al., 2017). The thalamus has also been proposed to integrate predictions from across disparate cortical areas, and subsequently compute the accuracy of those predictions (Grossberg and Versace, 2008; Bennett, 2020; George et al., 2020). The thalamus has further been suggested to operate as an attentional gate or searchlight (Crick and Koch, 1998).

The proposals in this article—for the role of higher-order thalamic nuclei—are not offered as exclusive proposals; rather, as a formal account of the computational anatomy of higher-order thalamic functions that complement or contextualize existing proposals. In what follows, we elaborate the relay picture of the thalamus in three important respects. First, following Mumford (1991), we propose that the thalamus acts as a blackboard, allowing different specialist cortical regions to cooperate in solving cognitive problems. Second, following the Bayesian brain hypothesis (Knill and Pouget, 2004; Doya, 2007; Rao, 2010)—and its expression *via* the Free Energy Principle (Friston, 2003)—we propose that in its blackboard role, the thalamus brings about a particular result—the aggregation, or summing, of the free energy contributions from diverse cortical regions, as they join in dynamic coalitions for active perceptual inference and planning. Third, we propose that the scarce bottleneck of thalamic processing is engaged particularly in novel tasks; and that thalamocortical circuits then train cortico-cortical links to take over those tasks as they become amortized and habitual. We hope that this thalamic blackboard picture may serve as an indicative framework to bring together the many interpretations of thalamic function which are now emerging. First, we describe—from first principles—what a blackboard is.

## Blackboard Architectures and Cortical Modules

The concept of a blackboard (Nii, 1986; Llinas and Anthony, 1993) has emerged over the past decades from computer science and artificial intelligence. A blackboard is a central store of information that can be addressed selectively (that is, written and read) by a set of independent computing processes, enabling them to solve problems cooperatively. In essence, one computing process may post partial information or hypotheses to a part of the blackboard, and other processes may retrieve that information selectively, enabling them all to work together to reach a solution.

The blackboard is an analogy to a group of human experts working cooperatively on a problem. Each expert may write an idea on any part of the blackboard. All the other experts can see what has been written and may contribute their own ideas. In this way, the group can collaborate to accomplish more than any single expert on their own.

In artificial intelligence, the computing processes may be accomplished by small independent expert systems, each one with limited knowledge. This was proposed by Erman et al. (1980) as an approach to sensor fusion. In scientific or commercial computing, the blackboard may be a computer database, such as a Relational Database (Date, 1976), and the computing processes may be independent computer applications with different users. In cognitive neuroscience, the computing processes may be different (i.e., functionally segregated) cortical regions, and the blackboard may be the thalamus which they all connect to (Zeki and Shipp, 1988). In the first instance, this proposal is consistent with the very widespread connectivity of the thalamus.

At any moment, to control its own movements, an animal needs to understand the locations and movements of things around it, inferred from its sensory data. The mammalian cortex has many cortical modules concerned with different kinds of sensory data, and with ways of analyzing sense-data, such as:

• Edge detection.
• Motion detection.
• Stereopsis.
• Sound location.
• Locations of touch and movement sensations.
• Shape from shading.
• Shape from motion.
• Linking data from two or more sense modalities.
• Recognition of learned shapes or movements.
• Knowledge of hierarchical structures, such as bodies and body parts.

We will consider these analytic attributes as cortical **knowledge sources** that are functionally segregated in different regions in the cortex (for instance, in visual or somatosensory maps) (Zeki and Shipp, 1988; Tononi et al., 1994; Friston and Buzsaki, 2016). It appears that many different types of knowledge source, and many different instances of some types, operate in parallel to maintain an internal model of an animal’s surroundings at any moment (Crick and Koch, 1998; Thomson and Bannister, 2003; Zikopoulos and Barbas, 2007; Saalmann and Kastner, 2009; Cruikshank et al., 2012; Lewis et al., 2015).

When it comes to recognizing an object with multiple features and its respective location, separate representations are encoding “what” and “where” in the visual hierarchy (Ungerleider and Haxby, 1994). From the perspective of Bayesian belief updating under the free-energy principle, these cortical knowledge sources can be seen as sets of marginal probability distributions (Friston et al., 2017a; Parr et al., 2020) that inherit from a factorization of probabilistic beliefs about the causes of sensation. For example, knowing what something is conditionally independent of where something is. In terms of Bayesian belief updating in the brain, the implicit factorization of probabilities implies the additivity of free energy gradients^1^ from different knowledge sources during multisensory integration. In this article, the role of the thalamus is taken to be the integration of free energy gradients from rapidly formed dynamic coalitions of knowledge sources. The modularity in question subsequently calls upon a gating mechanism that establishes the different types of interactions among these factorized representations. The ensuing proposal is then that the thalamus acts as a blackboard, though which inference based on different cortical knowledge sources are integrated into a Bayesian or posterior belief distribution over the causes of sensations. We will first focus on this integrative role—and then turn to the question: why are these Bayesian beliefs necessary for sentient behavior.

For any blackboard application—sensor fusion, computer databases, or sense data integration—there are some core requirements for the blackboard. First, the blackboard must be able to store information, if only for short periods, so that different knowledge sources can read and write information to the blackboard asynchronously. The reading and writing, in this context, corresponds to belief updating that could be mediated by variational message passing, belief propagation, or predictive coding; depending upon the nature of the generative model—and the particular way in which free energy gradients induce neuronal dynamics (Rao and Ballard, 1999; Bogacz, 2017; Friston et al., 2017b).

The suggestion that the thalamus, acting as a blackboard, needs to store information for short periods, is found in other formulations, such as Dumont and Aggleton (2013) and Warburton (2018), which implies that thalamic nuclei are also involved in longer-term memory. The complex roles of the thalamus imply that any thalamic nucleus may contribute to several functions; so short-term and long-term storage are not in conflict.

Second, the blackboard cannot just hold an unstructured heap of facts or hypotheses. Each knowledge source must be able to address its inferences selectively to some part of the blackboard. These inferences must be segregated on the blackboard, and knowledge sources must be able to selectively retrieve information from the blackboard. In short, the dynamic connectivity of the thalamus and thalamocortical connections must embody the generative model’s delicate causal architecture that is learned or distilled from the world.

These core requirements are implicit in the blackboard metaphor. A physical blackboard holds information as chalk marks, and these chalk marks are distributed across the plane of the blackboard so that different experts can read or write selectively to different parts of the blackboard. These requirements will be called the **addressing** requirements.

A blackboard architecture accommodates—simply and compellingly—the computational architectures required for Bayesian belief updating. In Bayesian inference, the posterior probability of some hypothesis (for instance, the probability that there is an object at some location in space) is proportional to its prior probability, multiplied by the likelihood that the hypothesis would have caused the current sensations. In variational treatments of Bayesian belief updating—of the sort implied by the free-energy principle—the posterior or conditional probability can be factorized to represent contributions from different knowledge sources, explaining conditionally independent aspects of sense data (e.g., *what* something is and *where* something is). This implies that the log probability of the hypothesis corresponds to the sum of log probabilities from different knowledge sources (modulo a constant for all hypotheses). Based on a physical analogy, this negative log probability can be expressed as Free-Energy, and the task of a brain in finding the most likely hypothesis is to minimize its free-energy or to minimize surprise inherent in sensory input. This minimization can be cast as a gradient flow on free energy, furnishing a straightforward description of neuronal dynamics (Friston et al., 2017a). Just as the log probabilities add, so do free-energy gradients.

This approach to cognition has a strong theoretical basis, because it can be shown, under very general conditions (Worden, 1995), that Bayesian cognition affords the greatest the fitness—and so is the target towards which the evolution of brains converges. Furthermore, the Bayesian brain can explain many different aspects of cognition (Knill and Pouget, 2004; Doya, 2007; Seth, 2015; Omidvarnia et al., 2017).

The ensuing Bayesian approach can be applied to a central problem in animal cognition, which impacts an animal’s survival at every moment of the day—the problem of inferring the locations of objects in peripersonal space from multi-modal sense data. The animal needs to know these locations at every moment to control its physical movement, from locomotion through to visual saccades. The application of Bayesian mechanics to the location of objects leads straightforwardly to a requirement for a blackboard architecture—where different knowledge sources are assimilated to furnish conditional probabilities for the spatial locations of objects.

The combination of blackboard architecture and Bayesian inference leads to a specific kind of blackboard—a **probability aggregator** (Worden, 2020b), where probabilities are factorized distributions encoding specialized representations, so logs of probabilities (and their gradients) are to be added, as in the Free Energy Principle (Friston, 2003; Parr et al., 2020). In the aggregator architecture, different hypotheses about the spatial locations of objects are segregated by location. For example, for any given hypothesis—that there is an object X at location Y—different knowledge sources estimate conditional probabilities for the hypothesis (based on different types of sense data) and post them to the blackboard. The blackboard aggregates (i.e., sums) the log contributions from the different knowledge sources. By Bayes’ theorem, this summation (when combined with a prior log probability) estimates the overall log probability of the location hypothesis, from the contributions of all the knowledge sources, aggregating information from different modalities and marginal representations. Maximizing the posterior or conditional log probability (i.e., minimizing the free energy) produces the most probable identity and location for each object, in the light of all the sensory evidence at hand.

To illustrate this principle: vision gives the animal a two-dimensional projection of its surroundings, encoded as 2-D locations of neurons in some retinotopically mapped visual cortex. To elaborate a three-dimensional model, as needed to control movement, various knowledge sources are applied (Leibo et al., 2015), such as:

1 Stereopsis.
2 Shape from shading.
3 Shape from motion.

According to varying circumstances, different knowledge sources may provide the most decisive depth information at different times, and they may confirm one another, or they may compete, inferring different depths. All this is encapsulated in a Bayesian estimate of depth from the different knowledge sources^2^. One way in which this could be computed is to sum the log probabilities of different depth hypotheses as an aggregate on a blackboard and to determine the most probable depth from the maximum of the sum—where the sum of the gradients in any variable is zero.

As a second illustration, consider the (multisensory) problem of integrating visual information and proprioceptive information, as is needed for instance in hand-eye coordination, or in paw-eye coordination for primates (Ernst and Banks, 2002). Proprioception gives information about the position of a limb, possibly through inferring joint angles; and vision gives conditionally independent information about the location of the same limb. At different times, one or the other knowledge source may dominate; but at all times, the best (fittest, most accurate) way to combine the two estimates—to estimate the location of the limb—is by Bayesian estimation. This can be done through the blackboard aggregation of log probabilities from the two knowledge sources. We will now unpack the implicit gating mechanisms in terms of attention and the pulvinar.

## Roles of Thalamic Nuclei

The role of the thalamus in cognition can be characterized by considering the roles of higher-order nuclei of the thalamus, including the pulvinar, the Mediodorsal (MD) nucleus, and the paraventricular thalamus (PVT). An interpretation of their roles is consistent with recent findings on the role of the MD nucleus in learning and memory for complex spatial configurations, and the PVT for balancing danger and reward.

## The Pulvinar

The pulvinar links mainly to posterior cortical regions involved in sense data processing, such as the visual cortex. This is consistent with a role for the pulvinar in mainly sensory processing: specifically, in building and maintaining a 3-D spatial model of “what is where” in the animal’s immediate surroundings, based on multi-modal sense data (see Rudrauf et al., 2017). This generative model of space underwrites Bayesian inference and learning—fitting to all sense data (except olfaction), using the blackboard/aggregator architecture.

In building a 3D generative model of the animal’s surroundings, attention to the most informative and precise inputs is important. Diverse evidence points to the pulvinar playing a role of this kind:

• There is a double dissociation effect in processing visual information whereby deactivating the lateral pulvinar suppresses V1 responses to visual stimuli, whereas superficial visual layers with overlapping receptive fields become more responsive as an effect of pulvinar activation (Purushothaman et al., 2012).
• The presence of presynaptic acetylcholine receptors in thalamocortical pathways (Lavine et al., 1997), known to modulate the gain of evoked responses in visual perception.
• Lesions to the pulvinar result in focal attention deficits (Snow et al., 2009).
• Neural activity in the pulvinar that is associated with task-relevant stimuli but not with distractors can be decoded, implying a filtering process (Strumpf et al., 2012; Saalman and Kastner, 2014) discuss how the Pulvinar regulates the flow of information between visual areas (Warburton, 2018), reviews the role of thalamic nuclei in object recognition tasks.

These examples and others (Shipp, 2004; Kanai et al., 2015) speak to a form of attentional selection mediated by the neuromodulatory effects of the type afforded by the pulvinar. The pulvinar is in the position to selectively enable pre-synaptic gain sensitivity to particular types of information. Note that the pulvinar is itself selecting the inputs that it aggregates. In other words, the pulvinar is, effectively, predicting the precision or weights that should be afforded the various knowledge sources: it is effectively selecting the kinds of knowledge sources that influence belief updating in a base optimal fashion.

In the Bayesian inference thought to be performed by animals or humans, the difficult problem is finding the optimal balance between the different types of sensory evidence and their implicit conditional probability distributions in relation to the different types of priors. That is, weighing the prior beliefs according to the sensory evidence sampled by the agent. This balance is mediated by the relative precisions (i.e., negative entropies) of the particular belief distributions in question. Deploying and mixing information is just as important to the agent as the information itself. The best candidate in maintaining this balance—by enabling specific representations (i.e., cortical knowledge sources)—is the pulvinar, inferring, and mediating visual attentional set.

### The Mediodorsal Nucleus

The MD nucleus is strongly linked to the prefrontal cortex, whose role relates to executive planning for actions. For non-human primates, this planning is largely the planning of complex spatial movements, asking questions such as “Can I reach that piece of fruit?” “Can I jump to that branch?” and “Is it strong enough to bear my weight?” There have been extensive investigations of the role of the MD nucleus in executive decision making, learning, and spatial cognition (Aggleton and Nelson, 2015; Mitchell, 2015; Parnaudeau et al., 2015; Ouhaz et al., 2018; Parnaudeau et al., 2018; Wolff and Vann, 2019), leading to a complex picture of many related roles for the MD nucleus. Here, we particularly address the need for spatial control of movement in tasks where MD-related deficits have been observed (Mitchell, 2015).

Movement-planning decisions depend on the 3-D configuration of objects in space, as represented in the pulvinar—but now require internal simulation of the planned movement—“If I jump, how far will I go?” and on memories of recent similar movements—“The last time I tried this….” These “what if” questions must be played out (possibly by imagining the movement at a declarative level) against the background of what is where now. That is the nature of planning. Then, when a satisfactory plan has been found, it must be carried out and monitored—another task requiring an accurate moment-to-moment model of what is where in three dimensions.

When we refer to “spatial cognition” or “spatial processing” in the context of the Pulvinar or the MD nucleus, we are referring specifically to a three-dimensional spatial model of the locations of objects immediately around the animal, as perceived and used to plan and control muscular movements, rather than to a two-dimensional navigational space. The latter space is linked to the hippocampus, to place cells and head direction cells, and the thalamic nuclei linked to the hippocampus, such as the anterior dorsal nucleus (Taube, 1995), and the ventral midline nuclei (Jung et al., 2019).

One might propose that the MD nucleus of the thalamus (and possibly other thalamic nuclei) uses a shared generative model of peripersonal space, as orchestrated in the pulvinar, to plan, to test in imagination, and then to execute, novel or complex movements, with the PFC.

This interpretation of the role of the MD nucleus is consistent with:

1. Its position in the thalamus, where it has access to the 3-D spatial model of current reality.
2. Its extensive cortical connections, particularly to the PFC with its executive role in planning.
3. Experimental findings on learning and memory for complex spatial configurations, in the presence of lesions to MD nucleus or PFC.
4. Its reciprocal connections with the supplementary motor area known for its contributions to movement control (Cunnington et al., 1996; Chen et al., 2010).
5. Recent theoretical formulations (Parr and Friston, 2018) suggesting that during inference, ascending messages from the MD nucleus to the motor cortex represent the free energy expected under each potential outcome, given the set of actions being considered.

Before comparison with the experimental findings, we outline the proposed mode of functioning of the MD nucleus in the setting:

• The PFC makes an executive decision that a certain goal needs to be achieved (e.g., grasping a piece of fruit, jumping across a stream). The goal requires a coordinated sequence of physical movements.
• If the goal or the circumstances are novel, it is necessary to plan and simulate the movements in three dimensions before carrying them out: failure in simulated 3-D space is cheaper than real failure.
• The MD nucleus acts as a blackboard for this simulation, using the model of objects in space computed from sense data in the pulvinar blackboard.
• The MD nucleus orchestrates cortical motion-control knowledge sources, which entertain possible movements.
• Planning may involve recalling memories of recent similar movements, for comparison.
• The action trajectory with the minimum expected free energy is selected.
• If PFC evaluates the likely level of success to be sufficient, the sequence is carried out—with pulvinar and MD nuclei acting as blackboards for the monitoring of the outcome: see “planning as inference” (Attias, 2003; Baker et al., 2009; Botvinick and Toussaint, 2012; Maisto et al., 2015; Kaplan and Friston, 2018).
• For novel sequences, cortical activity is coordinated through cortico-thalamocortical (CTC) driver pathways.
• If some sequence is repeated successfully, CTC driver activity produces plastic changes in the corresponding direct cortico-cortical pathways, *via* experience-dependent learning.
• After several successful repetitions, any movement sequence becomes “compiled” into direct cortico-cortical pathways, which are faster than the CTC path, and bypass the thalamic planning bottleneck.
• So successful planned movement sequences become habitual and unconscious, as the thalamus entrains cortex. This is learning to “infer” (Gershman and Niv, 2010; Series and Seitz, 2013).

This process is similar to the distinction in AI between “deep” or “causal” knowledge (such as explicit spatial modeling of movements), and “compiled” rules which are cheaper and faster to apply, once they have been compiled. In a similar vein, machine learning considers this habitation in terms of “amortization” (Rice and Barone, 2000; Zhang et al., 2018); namely, deferring the computational cost of planning by resorting to a hardwired habit (in the right context). The role of the thalamus is to do itself out of a job—to do the expensive central “bottleneck” work of explicit modeling of movements in space only when it is necessary, for novel challenges, consequently entraining direct cortico-cortical pathways to take over the job—which they can do in parallel, faster and without conscious involvement.

**Figure 1:**
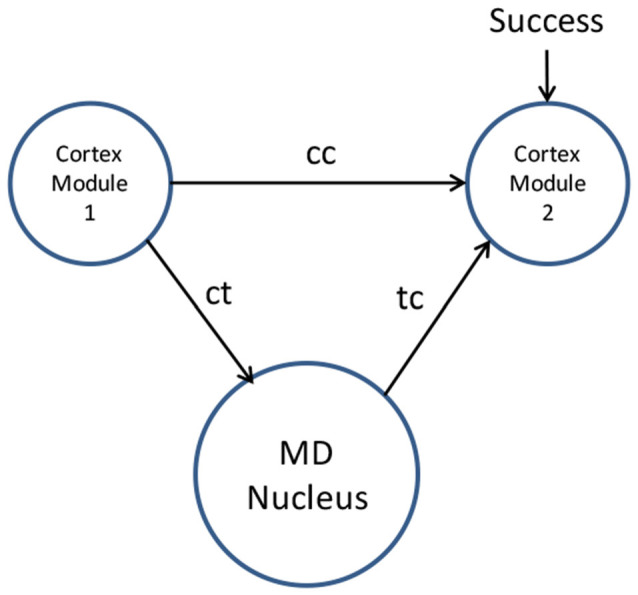
Mediodorsal (MD) nucleus training cortex by associative learning (i.e., “this is what I see myself doing in this situation”). Novel movements are coordinated by the MD nucleus relay driver neurons through corticothalamic paths (ct) followed by thalamocortical paths (tc). Repeated movements lead to synaptic changes facilitating a direct cortico-cortical path (cc) to cortex module 2. These cortico-cortical paths then manage the habitual movement without thalamic involvement.

The process of thalamus training cortex is illustrated in Figure 1.

We note that this suggestion, that the MD Nucleus trains the cortex in new movements, is not intended to be an exclusive account of what the MD nucleus does. As was noted in “Theories of Thalamic Function” section of the article, thalamic nuclei are involved in so many types of cognitive function that any one account of their function is bound to be incomplete. Different accounts, such as those cited in “Theories of Thalamic Function” section, can coexist.

Given the many studies of the role of the MD nucleus in learning and task performance, the evidence may sometimes seem contradictory. However, the overall picture seems to be that the MD nucleus is more involved in “rapid trial-by-trial associative learning and decision-making” (Mitchell, 2015) than in habitual tasks.

Heuristically, this process can also be understood by analogy to a SatNav (satellite navigation system). The first time any route is needed, the SatNav does an expensive spatial computation—comparing different routes for cost, time, and traffic, and so on. But if a route is selected and traveled, the spoken instructions are recorded—and the next time that route is needed, it is only necessary to replay the recording, with appropriate timings. Cortico-cortical links act as the recorder, and complex spatial computation is no longer needed. Only much more circumscribed planning (inference) is required—the cortex has learned to infer.

The notion that skilled habitual behavior is learned active inference could be compared with experimental results for spatial tasks with MD and PFC lesions. Potential neuroscience experiments best fitted to test this notion would use some task requiring the planning of spatial movements, in the following sequences:

a. Test novel—test habitual.
b. MD lesion—test novel—test habitual.
c. Test novel—MD Lesion—test habitual.
d. PFC lesion—test novel—test habitual.
e. Test novel—PFC Lesion—test habitual.

Here “test novel” tests the performance of the task when it is still new, and requires explicit planning of movements in space; while “test habitual” tests the same task when it has become familiar and routine. The key contrast is between sequences (b) and (c); (b) should show a greater impairment than (c), because in (c), the MD nucleus is no longer needed for habitual movements; while (d) and (e) should be more similar to each other than (b) and (c), because PFC lesions should affect novel and habitual movements comparably.

Several recent experiments have been sufficiently close to this design to shed light on the nature of spatial planning (Gaffan and Parker, 2000; Mitchell and Gaffan, 2008; Mitchell and Chakraborty, 2013). Those experiments lend support to the interpretation that the MD nucleus, by training the cortex, delegates the work and so does itself out of a job. In essence, these experiments suggest that lesions to the MD nucleus before training disrupt learning and performance; but lesions after training do not disrupt the performance of learned discrimination. These results are reviewed by Mitchell (2015) who writes: “*Recent evidence from monkey models of cognition shows that the magnocellular subdivision of the mediodorsal thalamus (MDmc) is more critical for learning new information than for retention of previously acquired information. Further, consistent evidence in animal models shows the mediodorsal thalamus (MD) contributes to adaptive decision-making.”*

**Figure 2:**
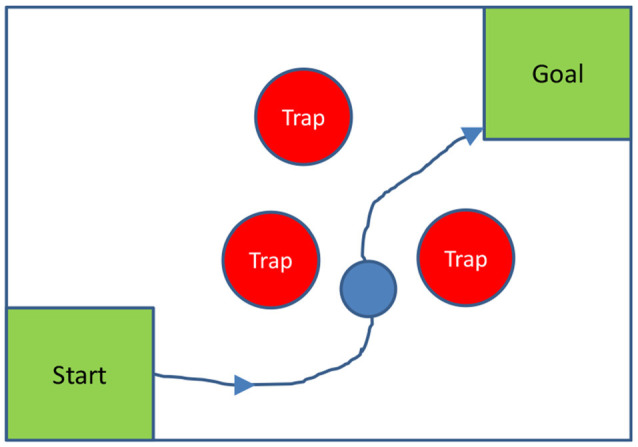
Two-dimensional spatial planning task: a monkey is required to guide a ball to a goal, by moving a mouse, without touching any trap. In the diagram, traps are stationary; but they could also move, to make the planning task harder.

To more fully test the hypothesis that the MD nucleus is used to plan movements, and subsequently, MD entraining cortex in the planned movements, similar experiments could be done on non-human primates with MD functioning temporarily inhibited, for instance using optogenetic suppression (Rikye et al., 2018), using a task with explicit planning of movements; for instance, requiring monkeys to move an object to a goal around various obstacles or traps. A two-dimensional version of this test could be configured with a computer screen and a mouse-guided ball, as shown in Figure 2 below.

Experiments using these sorts of tasks could explore not only the role of the MD nucleus but also the number of examples needed to train the cortex and make a task habitual (as measured by reduced task completion times).

### The Paraventricular Nucleus

A key aspect of animal behavior, with a strong influence on survival, is making decisions that balance danger against reward—for instance, knowing whether to feed or flee, explore or exploit (Cohen et al., 2007; Humphries and Prescott, 2010; Humphries et al., 2012; Friston and Buzsaki, 2016; Konig and Buffalo, 2016). In rodents, the thalamic paraventricular nucleus is known to be involved in these kinds of decisions (Choi and McNally, 2017; Choi et al., 2019).

In a free-energy formulation of a risk-reward trade-off, there needs to be some common currency in which both risk and reward are represented, to ensure a seamless trade-off. The common currency is a logarithm of the probability of survival; diverse risks, such as predation, reduce this logarithm, and rewards such as food increase it. In any situation, there is a diverse set of risks, and the probabilities of death from the different risks can usually be multiplied (factorization); therefore the (expected) free energies can be added.

This calculation is therefore suitable for an aggregator architecture, adding together the free energies from a dynamic coalition of knowledge sources, much as we propose the pulvinar sums free energies in spatial perception and movement planning. Crucially, for the risk-reward trade-off, as there are many potential risks, and a few of them are significant at any time, free energies from a dynamic coalition of knowledge sources need to be summed—so hard-wired cortico-cortical connections are less suitable to do the sum, and a central blackboard/aggregator is a more suitable architecture. This is consistent with the known role of the paraventricular nucleus in these trade-offs, and a blackboard role for the thalamus.

Based on the result for motion control (cited in “The Mediodorsal Nucleus” section) that the thalamus is no longer involved when a motion becomes habitual (e.g., Mitchell, 2015), and our interpretation that the thalamus “trains” cortico-cortical circuits to perform habitual movements, we might venture a similar prediction for risk-reward behavior. While Choi et al. (2019) have shown that temporary suppression of the paraventricular nucleus by chemogenetics disrupts risk-reward trade-offs when they are novel, we would predict that when some trade-off becomes routine, suppression of paraventricular thalamus, in a similar manner to that investigated by Choi et al. (2019) would no longer disrupt it. Testing this prediction would depend on some risk becoming routine.

## How Is The Blackboard Addressed?

The examples of depth perception and multi-sensory integration illustrate how hypotheses from different knowledge sources need to be combined to support Bayesian inference in scene construction (Hassabis and Maguire, 2007; Mirza et al., 2016). Stereopsis, shape from motion (Murray et al., 2003), and shape from shading can each operate across a large part of the visual field. Further, the varying spatial location has been shown to impact perceptions of the same object (Finlayson et al., 2020) and therefore to impact which relevant cortical knowledge sources would be dynamically engaged. There can be many instances of each type of knowledge source operating in parallel, at different parts of the visual field. How are their probabilities to be aggregated? Which instance of shape from motion should be aggregated with which instance of stereopsis?

There is one possible answer to these questions. Two knowledge source instances can only be aggregated (i.e., their log probabilities should be added) if they refer to the same latent causes—that is, to a thing at the same inferred location. Therefore, hypotheses on the blackboard need to be segregated by location—to instantiate the prior belief that two things cannot occupy the same location in space. For features in the animal’s immediate surroundings (including its own limbs), segregation by hypothesis implies segregation by location. This question, of the appropriate combination of information from different cortical knowledge sources, has also been cast as the binding problem—how to bind together the activities of different cortical modules (Treisman and Gelade, 1980; Tononi et al., 1994; Treisman, 1998; Fingelkurts et al., 2010; Feldman, 2013). The current analysis suggests that a possible solution to the binding problem is binding by inferred location^3^, through the blackboard.

There is a further important requirement for Bayesian inference about objects in space. There is an important prior probability that in an allocentric frame of reference, most of the things surrounding the animal do not move. This prior is so universal that it can be represented and used in animal brains, for two related purposes: first, if something is known with high confidence to be static, the animal does not need to keep checking its location. Second, something which moves deserves attention. This would involve mandating attention appropriately: anteriorly to the PFC and motor cortex *via* the MD thalamus and posteriorly *via* the pulvinar (Feldman and Friston, 2010; Brown et al., 2011; Vossel et al., 2015; Parr and Friston, 2017; Mirza et al., 2019) but will not be explored further in this article.

So far, we have discussed the requirement for segregation of information in the blackboard at Marr’s (Marr, 1982) level 2, of algorithms and data structures. The question arises: how can segregation by location be implemented at Marr’s level 3 of neural implementation? We shall approach neural levels in three steps. Among many possible neural implementations, it is worth picking out two extremes, namely, “focal” and “distributed” representations.

In a **focal** neural implementation, one location in real space may be represented by the firing of a few neurons at a specific location in the brain. This is the kind of representation used in the V1 visual cortex and used in somatosensory maps in the brain. The focal representation of the V1 cortex is replicated in the thalamic LGN, which connects to V1 by topographically organized relay neurons. In secondary thalamic nuclei such as the MD nucleus and the pulvinar, the situation is less clear. While there are two concentric visual maps in the pulvinar, their relation to cortical maps is a much more “blurred” (i.e., distributed) relationship (Shipp, 2003).

In a **distributed** representation of space, one point in space is represented not by the enhanced firing of neurons clustered at some location in the brain, but by a pattern of firing across many neurons. One can illustrate a distributed representation in a single dimension, by the example of a Fourier representation. If there is a set of neurons arranged along a single dimension in the brain (called x), then a pattern of firing rates R ~ [1 + cos (kx)] across those neurons can represent an object at location k. If there are many neurons arranged along the dimension x, then their firing rates can simultaneously represent the locations k, k′, and so on of many objects, by superposition of the different cos (kx) patterns of firing rates.

Such a Fourier-like representation is capable of high capacity and high spatial precision (as is required to segregate information on the blackboard); if many neurons (or synapses) with different internal position x are involved in representing one wave-like cos (kx) pattern, then k can be determined to high precision. One output neuron can have its input synapses spatially distributed as cos (kx), and so be preferentially sensitive to 1 value of k, with high selectivity.

The Fourier representation generalizes to three dimensions, provided that neurons and synapses are extended in three dimensions (as they are in the thalamus), and not in a 2-D sheet, as in cortex. That is, a represented position **k** can be a three-vector; then the distribution of firing rates can be [1 + cos(**k.x**)], a wave-like distribution in the volume of the thalamus, representing positions with high precision in three dimensions. Because the output neurons can be selective in **k**, the same set of neurons can, by superposition of firing rates, represent the positions of many objects (different **k** values) simultaneously.

The Fourier representation of position information is only given as one example of a distributed representation, but it seems to be a powerful and instructive example. It shows how, in a three-dimensional volume of the thalamus, a distributed neural representation could give three-dimensional spatial segregation of information with high precision and high capacity—which is one requirement for a blackboard role. It is notable that each thalamus, unlike many parts of the brain, has an approximately spherical shape, with comparable extension in all three dimensions, so enabling it to store positions with high precision in three dimensions, using a distributed representation. This regular shape of the thalamus is preserved across many species (Jones, 2007).

## Spatial Steering of Sense Data

Small regions or modules of the cortex can be classified along a spectrum between two extremes:

1. Regions dedicated to a particular patch of incoming sense-data, such as parts of the V1 visual cortex, or of somatosensory cortex; these regions are typically parts of sensory maps, and perform some homogeneous function across the map, such as edge detection.
2. Regions dedicated to a particular function, such as face recognition or word recognition, may use sensory data from many different sources and locations.

There are regions of cortex between the two ends of the spectrum—for instance in higher visual areas concerned with inferring shapes and form (Zeki and Shipp, 1988; Lueck et al., 1989). This characterization of the cortex—by such a spectrum—may be a simplification, but it serves to define a lower limit to the diversity of cortical regions to which the thalamus is connected.

We focus on the second end of the spectrum, noting that a face recognition module needs to learn and recognize faces across a large part of the visual field. We also note an insight from building artificial neural nets. It is possible to build a working neural net for face recognition; but to make it learn faces in the shortest possible time, all the faces must be properly aligned on some input grid of the net (Denker et al., 1987). Variable alignment leads to much slower learning. The requirement for spatial alignment extends to hierarchical multi-layer “deep” nets (LeCun et al., 2015). If there is a “nose recognition module” serving the face recognition module, the output of the nose recognition module needs to be properly aligned as an input to the face recognition module. That is, the nose needs to be at the center of the face.

This leads to a requirement for **spatial steering** of sense-data between cortical modules. In some models of hierarchical pattern recognition (Olshausen et al., 1993, 1995; Lee and Mumford, 2003), spatial steering is accomplished by direct cortico-cortical connections. The spatial steering needs to be rather precise. Consider recognizing a face 10 m away, and the need to align the recognized nose properly on the face. Absolute displacements from the animal, rather than relative displacements within an object need to be aligned (Worden, 2020b). If precise alignment is done by the selection of alternative cortico-cortical fiber bundles, it may require prohibitive numbers of bundles, most of which are idle most of the time. In machine learning and some treatments of spatial attention, this steering is cast as attentional orientation or selection; e.g., see Humphreys et al. (2009).

Since a thalamic blackboard needs to segregate information by spatial location, there is an alternative way to do the spatial steering of sense-data needed for hierarchical pattern recognition. If the “nose recognizer” posts its results to an inferred location (an address in the blackboard), and the face recognizer reads (as it must) from the same location, the combination of reading and writing has **steered** the sense-data spatially between these two cortical modules. Segregation of information on the blackboard, together with selective writing and reading, implies steering of sense-data. Spatial steering of sense-data is one of the core functions of the thalamus as a blackboard.

This leads to a more powerful concept of the thalamic “gating” of information. Gating is often conceived as the thalamus simply switching neural paths on and off; a kind of simple on-off filtering, or tuning up and down of arousal. But it can also be seen as a precise spatial routing of sense data and neuronal message passing between cortical modules. This is a much more demanding requirement than simple gating. It is a requirement for signal processing rather than for computation. If the thalamus does spatial steering of sensory information, how might it do it? Distributed representations—in particular, Fourier representations—are again instructive.

**Figure 3:**
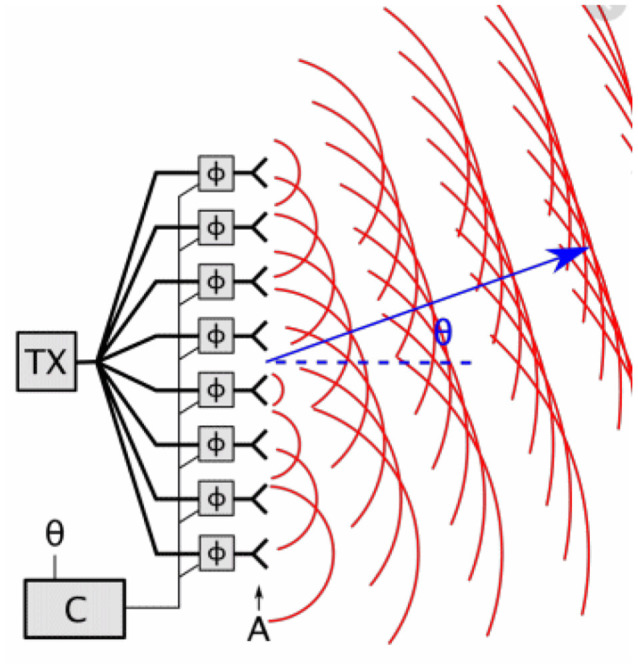
The use of a phased array antenna, where the relative phase of the signals emitted by neighboring antennae controls the direction of the signal emitted.

An analogy from engineering—to illustrate this—is a **steerable phased antenna array** used, for instance, in satellite communications. For steering in one dimension, this is a regular linear array of small antennae, all transmitting at the same frequency (and therefore at the same wavelength). If the antennae are all in phase, then the radiated signal has a strong peak at 900 from the line of the array, where they all interfere constructively. However, if there is a phase lag between each antenna and the next one, the peak of constructive interference is moved away from 900. By controlling the relative phases of the antennae, the output signal can be spatially steered in different directions. This is illustrated in Figure 3.

This illustrates how a time lag between each antenna and the next one, which is less than one cycle of their oscillations, means that the wavefronts are all in phase at some angle θ to the vertical, where θ is determined by the time lag.

The steerable array principle could be applied in the thalamus, as follows:

• The phase of the neural signals is the phase of firing relative to some prominent thalamocortical rhythm, for instance, an alpha rhythm at 10 Hz, with a 100 ms cycle time.
• The relative phase of different neurons or synapses is controlled by introducing time lags in the range of 0–100 ms.
• Locations are represented in a Fourier representation, with three-dimensional wave vectors **k**.
• Each thalamic relay neuron has a wave-like spatial distribution of input synapses cos (**k.x**) over some region of positions **x** in the thalamus.
• The represented position **k** is tuned by varying the time lags on input synapses in the 0–100 ms range, in a way dependent on the position of the synapse in the brain.

With the Active Inference framework in mind, this type of attentional modulation would be mediated by nonlinear synaptic mechanisms of the sort reviewed above—and implicated in the deployment of precision. The basic idea is that to compute the posterior estimate of spatial location, the log-prior and log-likelihood would each be multiplied by their respective precisions (represented in the thalamus) then summed together. In this case, information *per se* is not affected since the logs themselves, which are provided by the cortex, are not being modified. However, the message passing is affected—messages being switched on or off—depending upon the afforded precisions.

This is only a high-level sketch of how the thalamus could steer sense data, and many details remain to be resolved. However, even based on this high-level sketch we can start to compare the requisite computational architecture with thalamic neuroanatomy.

## Thalamic Neuro-Anatomy for Steering and Aggregation

The previous sections have examined the hypothesis of the thalamus as blackboard at Marr’s (1982) Level 2, of algorithms and data structures. How does this description map onto thalamic neuro-anatomy, at Marr’s Level 3? The hypothesis can be related to several prominent features of thalamic neuroanatomy and physiology, including:

• Quasi-independent thalamic relay cells.
• Thalamo-cortical rhythms.
• Diffuse cortical connectivity of higher-order thalamic nuclei.
• Inhibitory interneurons.
• The regular three-dimensional shape of the thalamus.
• Driver and modulator pathways.
• Triadic synapses.

The following discussion applies mainly to higher-order thalamic nuclei such as the MD nucleus and the Pulvinar, rather than first-order nuclei such as LGN (which appears to have only a minor spatial steering function). Higher-order nuclei occupy most of the volume of the thalamus. In contrast to neurons in the cortex, thalamic relay neurons have no local excitatory connections. Is this distinctive feature consistent with a blackboard role?

In what follows, we use the term “relay neuron” purely to describe a type of neuron which is prominent in all thalamic nuclei (except the TRN)—without implying that the function of any nucleus is only a “relay” function.

A lack of local recurrent excitatory connections implies that it is not possible to sustain some pattern of neural firing by local positive feedback. This limits the ability of the thalamus to complex computations or to carry out one blackboard function—short-term memory for example—using only local connections within the thalamus. However, hypotheses can be sustained over unlimited periods by positive feedback between the thalamus and cortex, for instance in a 10 Hz alpha rhythm. This cycle can not only sustain short-term memory; it can also support a near-Bayesian optimal fit of hypotheses and sense data, as described in Worden (2020b). The lack of local excitatory connections in the thalamus does not rule out a blackboard role. It is known (O’Reilly et al., 2020) that a 10 Hz alpha rhythm is specifically associated with layer 5 neurons in the cortex and to the pulvinar.

Relay neurons in thalamic nuclei can support segregation by location in a distributed representation, as described in the previous section. For a distributed representation to give good separation in all three spatial dimensions, the neurons must be extended in all three dimensions—as is done by the approximately regular three-dimensional shape of the thalamus. In a distributed representation, segregation is not by relay neurons, but by patterns of firing across many relay neurons. In this connection, there may be a role for inhibitory interneurons.

In many signal processing applications, the linearity of transducers is required. This kind of fidelity in the message passing may be important in the thalamus for two reasons:

1. As an example of a distributed representation, the Fourier representation depends on linearity, in the following sense: if a point at position **k** is represented by a firing pattern [1 + cos (**k.x**)], a non-linear transform of this pattern (a harmonic distortion) would lead to higher harmonics like cos (2 **k.x**) and so on—producing spurious represented objects at positions 2 **k**, 3 **k** and so on. The linearity of the transducers will minimize the occurrence of such spurious “ghost” traces.
2. The overall probability of a hypothesis, evaluated from all relevant knowledge sources, is the one with maximum model evidence with weighted factors summed over all sources, with different sums for different distributions across the distributed representation. For the hypothesis with maximum model evidence to be found, the summation needs to be as close to linear as possible.

Seen as a transducer or amplifier, a neuron is not typically expected to be highly linear. A relay neuron on its own is expected to introduce non-linear distortion. However, inhibitory interneurons, which are a prominent feature of the thalamus, may play a role here.

Again, if we recourse to engineering analogies, the design of an **operational amplifier** uses negative feedback (through resistors) to convert a high-gain, non-linear amplifier into a lower-gain, but highly linear amplifier. In the same way, local negative feedback by inhibitory interneurons could convert the non-linear high amplification of a relay neuron into a more linear amplification—which is better suited to carry a factorized representation and to sum log-likelihoods for Bayesian maximum marginal likelihood estimation.

Next, consider the spatial steering function of the blackboard. To serve any signal steering function, the blackboard/thalamus must have two distinct types of inputs. These are the signal being steered (i.e., information *per se*) and the instructions about where and how to steer it. A prominent feature of thalamic neuroanatomy is the distinction between driver and modulator pathways (Sherman and Guillery, 2006). This two-way distinction could be linked to the distinction between signal and steering instructions, and could even be the same distinction.

Next consider the mechanism for spatial steering, using a distributed neural representation, and neural firing phased relative to a thalamocortical “carrier” frequency (e.g., at 10 Hz). As in a phased antenna array, signal steering can be accomplished by introducing a controllable phase shift within the 100 ms cycle. To tune a given relay neuron to be sensitive to a region in inferred location **k**, different phase shifts would need to be applied to different input synapses of the neuron, depending on their location in the thalamus. In this way, thalamocortical connections to a given cortical module could all be sensitive to a small region around some position **k_0_**, with the center **k_0_** of the region of attention being tuneable within the thalamus.

A distinctive feature of the thalamus is the presence of triadic synapses in glomeruli (Sherman and Guillery, 2006, 2011), where three or more neural inputs converge in one synaptic structure. This contrasts with the more usual dyadic input-output relation between two neurons and supports the convergence of two or more neural inputs—such as a sensory signal and its steering control.

Triadic synapses could have the function of introducing a controllable time delay (a phase shift), in the region 0–100 ms, to give spatial steering of a distributed neural representation. As an alternative to controllable delays, the use of sigma-pi neurons for signal steering is discussed in (Worden, 2020b). Triadic synapses could play a sigma-pi role in the thalamus.

These proposals for steering mechanisms are most relevant to secondary thalamic nuclei such as the pulvinar, rather than to primary nuclei such as LGN, which seem to support a more map-like relay function. The secondary nuclei are amongst the largest in the thalamus and have diffuse cortical connections, consistent with spatial steering to diverse cortical knowledge sources.

While the pulvinar is largely concerned with orchestrating sense-data from the posterior cortex, including vision, the other large secondary thalamic nucleus, the MD nucleus, is more closely linked with higher-level decision-making functions in the prefrontal cortex (Mitchell et al., 2014; Mitchell, 2015; Dehghani and Wimmer, 2018). The MD nucleus may be involved in motor and proprioceptive inference (Friston et al., 2015, 2016), where 3-D movement creates new possibilities and new options to compare. For non-human primates, decision-making is largely deciding about physical movement in space and time. The control of motion, like the perception of objects in local space from sense data, is intimately linked with the representation of three-dimensional space. So it is reasonable to expect that the MD nucleus, like the pulvinar, is concerned with the spatial segregation and spatial steering of information—both sense-data and motor commands.

It appears that in many ways, the neuroanatomy and physiology of the thalamus may be consistent with a blackboard/aggregator function, including spatial segregation and steering of sense data and processed sense data. The match appears to be good, and it can lead to suggestions for further experimentation and modeling, providing informed predictions for detailed tests and models of a thalamic blackboard/spatial steering function.

In the context of the interactions between the thalamus and the cortex, several additional observations are consistent with the idea that the thalamus operates as a blackboard whereby hypotheses from cortical knowledge sources are aggregated, exchanging information in an aggregator cycle (Worden, 2020b) between the thalamus and cortex:

• Cortex and thalamus oscillate on the same alpha cycle (Lörincz et al., 2009; Hughes et al., 2011).
• Cortical columns appear to oscillate between superficial layers “on” while deep layers “off” (Lörincz et al., 2015; Pluta et al., 2015; Naka and Adesnik, 2016).
• Layer 6 corticothalamic neurons provide modulatory input to thalamic relay neurons that project to the same column (Reichova and Sherman, 2004; Thomson, 2010; Sherman, 2017).
• Relay neurons only burst if they get this modulatory input (Jahnsen and Llinás, 1984; Hughes et al., 1999; Sherman, 2001; Guillery and Sherman, 2002).

**Figure 4:**
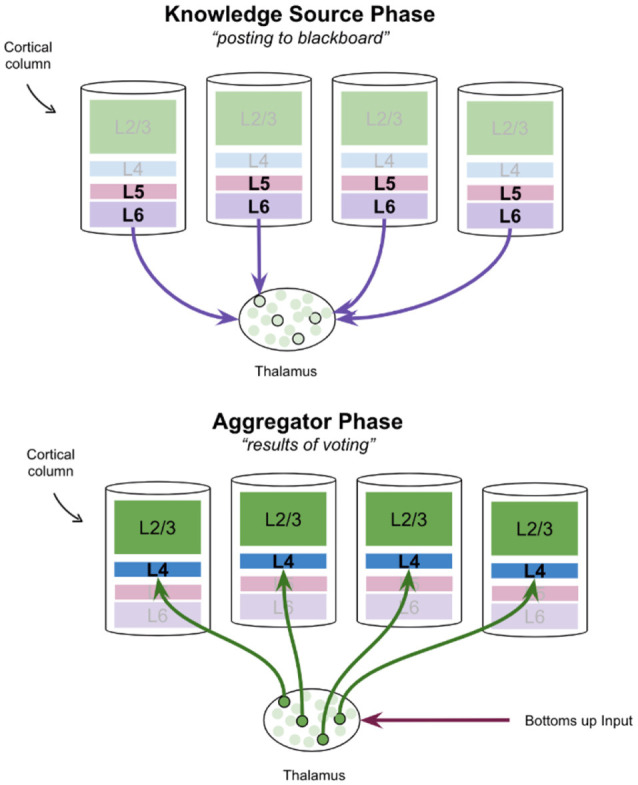
A proposal for how the thalamus may aggregate votes from cortical knowledge sources, and then post results back to those knowledge sources, in two successive phases. Note that in the frontal cortex there is no layer 4, but instead, thalamic relay neurons synapse directly onto pyramidal cells in L2/3.

The alpha cycle may be a mechanism by which cortical knowledge sources communicate with the thalamic blackboard. It has been proposed that entire thalamocortical networks oscillate between a “knowledge source phase” and an “aggregator phase” (Worden, 2020b). In this interpretation, the deep layers of cortical columns, specifically layer 6, post hypotheses to the thalamic blackboard on the “knowledge source phase.” Once these hypotheses are integrated, the results of this process are posted back to cortical columns during the “aggregator phase.” This is shown in Figure 4. Hypotheses that are *consistent* with bottoms-up input will be reinforced as only relay neurons with both modulatory and driving input will burst fire (Jahnsen and Llinás, 1984; Hughes et al., 1999; Sherman, 2001; Guillery and Sherman, 2002). This proposal of thalamus function has remarkable consistencies with various theories of cortical columns which have attributed a “voting” process to the modulatory input from layer 6 corticothalamic neurons to thalamic relay neurons (Grossberg and Versace, 2008; Bennett, 2020).

If knowledge sources post “what is where” to the thalamic blackboard, then this suggests that a given knowledge source either: (a) contains representations of different locations in space (e.g., a nose detector that can detect noses in multiple locations in space) or (b) a given feature (e.g., “nose”) is duplicated across multiple knowledge sources, each with separate spatial receptive fields (e.g., multiple nose detectors, for detecting noses in different locations in the visual field). Interestingly, recent theories of cortical columns corroborate both ideas whereby a given column contains spatial representations, and features are duplicated across columns. Specifically, Hawkins’ “Thousand Brains Theory” of intelligence (Hawkins et al., 2019) proposes that each cortical column generates a complete model of the world. He suggests that each column builds a complete 3D map of objects/features across a broad receptive field, as opposed to only representing a specific feature at a specific location. Hawkins goes on to propose that “grid-like” cells within each cortical column represent a location in space, which he suggests exists in layer 6. The thalamic blackboard proposal can be interpreted as an application of Hawkins’ theory, whereby these redundant and overlapping models of the world can be integrated and disambiguated with each other through oscillatory phases with the thalamus.

This is an unsettled topic in which proposals are still fluid. In contrast to Hawkins’ proposals, we note the suggestion in Worden (2020b) that cortical knowledge sources need only store and manipulate small **relative** displacements between features of an object, reducing their need for high spatial precision.

Cortical columns or modules have also been interpreted as simply *factors* of the Bayesian beliefs held by a system (Parr et al., 2020). It is commonly assumed that posterior beliefs are independent of each other, and hence factorizable, this is known as the “mean-field approximation” that renders Bayesian inference tractable in the form of variational Bayes or approximate Bayesian inference. This kind of inference could plausibly be implemented in the brain, as described above (Parr et al., 2020).

## Thalamic Neuro-Anatomy to Enable Cortical Learning

In this section, we discuss how thalamocortical neuroanatomy might support the second key function of the thalamus proposed in this article—which is to train direct cortico-cortical pathways, to do itself out of a job.

As shown in computational models of variational inference, computing the posterior requires a separate optimization for each data point to compute the best fit variational posterior (Kim et al., 2018). In other words, an inference can be computationally expensive and slow as it scales linearly with the amount of data. A computational technique called “amortized inference” dramatically improves the speed of inference by instead training a neural network to learn the mapping between observations and variational parameters (Gershman and Goodman, 2014; Marino et al., 2018). In other words, *learning* to infer. This process “amortizes” (or distributes) the computational cost of inference over many observations, as opposed to redoing this optimization each time. Through this lens, thalamocortical networks can be thought of as training corticocortical networks to infer on their own.

To describe this process, we consider the involvement of the MD nucleus in a typical movement planning task, which is to reach out and grasp a piece of fruit. In considering this task, it is worth recalling the analogy of the recording SatNav—which for the first use of a route, does complex spatial planning; and for later uses, simply replays the recording.

Note that any movement planning task has a natural hierarchical structure—where the hierarchy is a (time*limb) hierarchy. To grasp a piece of fruit, the task has four stages:

1. **Arm move**: Move the arm so that the hand is in the right place.
2. **Hand grasp**: Pick the fruit.
3. **Hand to mouth**: Move the fruit to the mouth.
4. **Eat fruit**: Chew.

Each step must be started approximately when the previous step has been completed—so the steps form a natural hierarchy (of depth 1) in time. A more realistic deeper hierarchy could involve sub-steps within steps, and parallel movements for within a step different body parts, such as fingers. The full movement involves the arm, hand, fingers, and mouth—so will involve several distinct regions of the cortex (suppose that cortical modules A, B, C, D carry out steps 1, 2, 3, 4 of the sequence). This is consistent with the extensive thalamocortical connections of the MD nucleus.

The hierarchical structure breaks the task down into steps that may be learned at different times. In this case, both the “hand grasp” and “eat fruit” steps would have been learned previously (i.e., recorded by cortical modules B and D), and the new learning task is just to replay B and D when needed, to “string them together” with novel arm movements from modules A and C. A hierarchical breakdown of the learning task is usually essential to narrow down the space of learnable patterns, to make learning feasible from small numbers of learning examples.

The first time that the novel arm movement (1) is made, cortical module A records it. On completion of (1), the previously learned grasping movement (2) is re-played by a cortical module B. This replay sequence is initially coordinated through cortico-thalamo-cortical (CTC) pathways. Each time the sequence is replayed successfully, there are plastic changes in the direct cortico-cortical links so that they can soon take over the job—it becomes habitual—and the CTC pathways are no longer needed. The movement becomes a top-down sequential cascade of cortical modules, each one replaying its learned sequence of movements (possibly by triggering sub-movements) and then triggering the next cortical module (Haruno et al., 2003; George and Hawkins, 2009; Maisto et al., 2015; Rikhye et al., 2019).

## Relations to Existing Work

There is a huge body of experimental work on thalamic neuroanatomy and neurophysiology to which this article is indebted, and which has not been fully referenced. For work before 2007, we have relied on the definitive books by Sherman and Guillery (2006) and Jones (2007) and references therein.

Since the ideas of this article about spatial segregation and steering of sense data are mainly applicable to higher-order thalamic nuclei such as the MD nucleus and the pulvinar, we have focused on articles directly relevant to them, including Shipp (2003), Sherman and Guillery (2013), Mitchell et al. (2014), Mitchell (2015), and Usrey and Sherman (2019). Similarly, work on thalamocortical connectivity (Behrens et al., 2003) has been used for insights into the connectivity of higher-order thalamic nuclei such as the pulvinar.

Beyond that, this article has been influenced by works that abstract general architectural or computational principles about the thalamus, such as Sherman (2016), Nakajima and Halassa (2017), and Halassa and Sherman (2019). The thalamic circuit motifs explored by (Halassa and Sherman, 2019) are particularly relevant—for instance, motifs involving triadic synapses, or other motifs that can be mapped onto the thalamus-as-blackboard concept in ways that remain to be explored.

The blackboard notion has been investigated by several authors, notably Baars (1988), Mumford (1991), O’Reilly et al. (2017, 2020), and Dehghani and Wimmer (2018). This article links these ideas to Bayesian inference, notably the Free Energy Principle of Friston (2003).

The computational model of O’Reilly et al. (2017, 2020) is relevant to this article, since it shares several important features, yet has key differences. Like the model of this article, their model hinges on the pulvinar in a blackboard role, and on a cortico-pulvinar alpha rhythm. However, the two models use these ingredients for different purposes. The main difference is that the model of O’Reilly and colleagues is largely a model of learning. Learning is typically a process that takes place over longer timescales (days or weeks); whereas this article also addresses a more basic, pre-learning question: how does the thalamus contribute to immediate spatial cognition on sub-second timescales?

While O’Reilly and colleagues interpret the 10 Hz cortico-pulvinar alpha rhythm as supporting a predictive learning process, predicting over the next fraction of a second, but driving synaptic changes which take place over days or weeks, in this article the same 10 Hz rhythm defines a phase for spatial steering of processed sense-data. Steering sense-data is a pre-learning process, operating at time scales of tens to 150 ms, and it provides the input data for cortical knowledge sources such as shape from shading, stereopsis, or shape from motion, which need involve no learning. Spatial steering is required for perceiving the 3-D shapes of irregular rocks or terrain or plants, simply for locomotion, before any question arises of learning how to classify objects by their shapes—as in the O’Reilly model. Therefore the two models both depend on cortico-pulvinar alpha rhythms but propose different interpretations of what those rhythms do. These proposals may even co-exist. This may pave the way for productive experimental studies.

Although this article has concentrated on spatial cognition as the main example to exhibit the type of orchestration, gating, and steering implied by thalamic architecture, another key theme is that of balancing prior preferences (rewards as encoded by the agent) and exploratory drive. In the context of decision-making, planning as inference, and learning to infer, the thalamus can be seen as a purveyor of precision. More specifically, the MD nucleus would have the vitally important role of assigning appropriate precisions to simulated future consequences of particular behaviors and evaluating these according to the agent’s prior preferences. This hypothesis is in line with research in neuroanatomical connectivity between the PFC and the MD (Funahashi, 2013), and its putative consequences in both working memory and how it is used by thalamic nuclei. A basic idea formulated using the Active inference framework would be that there is a comparison in natural units (nats) between the log probability of information gain given a particular prior preference and the log prior preference itself, thus allowing the agent to decide whether to explore or exploit (Parr and Friston, 2018; Da Costa et al., 2020). This aspect of goal-directed behavior, and the role MD nucleus plays in reward evaluation as well as in the explore-exploit trade-off—remains to be explored. There are links to the role of the paraventricular thalamus in the risk-reward trade-off, discussed in this article.

Part of the territory of this article has been explored in more computational detail, with less emphasis on thalamic neuro-anatomy and with greater emphasis on the scaling, speed, and precision achievable by a blackboard/aggregator architecture, in (Worden, 2020b). Some key results in that article, relevant to this article, are:

1. In terms of required cortical connectivity and its energy costs, the hub-and-spoke architecture of a blackboard is much more efficient than a fully distributed cortico-cortical architecture.
2. The required spatial steering of hypotheses (for instance, in hierarchical pattern recognition) must be steering in absolute positions relative to the animal—not just relative positions within an object. This places a high requirement on the precisions underlying signal steering.
3. Spatial steering involves the accurate computation of spatial displacements; this is 3-vector subtraction, and it can be done with high precision and fast in a distributed Fourier representation.
4. The spatial steering function and its implied neuromodulation is more efficiently done in the central blackboard/aggregator, than separately in each cortical knowledge source. The latter approach would require massive replication of the steering functionality.

This article has said little about the issue of object constancy in an allocentric frame of reference, and how an aggregator architecture might exploit that important prior probability. The lack of local recurrent excitatory connections in the thalamus, preventing local persistence by positive feedback, seems to underline that problem. Worden (2020a) investigates a radical solution—that as well as neural synaptic connectivity, there is a physical wave excitation in the thalamus, which serves as a short-term memory for spatial information in a Fourier-like representation. The considerations of this article, about the neural implementation of the aggregator function, apply whether or not that more radical suggestion of a wave excitation in the thalamus is correct.

Finally, the preservation of the thalamic architecture across mammalian species, and many others (Sherman and Guillery, 2006; Jones, 2007) seems to point to an early evolutionary origin and a universal functional role. It is worth noting that a requirement for precise spatial steering of sense-data has existed since the first compound eyes evolved, with up to 10,000 receptors, in the Cambrian period (Parker, 2003). There would be little point for a Cambrian animal to have a high-resolution vision if its brain cannot precisely steer the signal to specialized processors. Precise spatial steering and Bayesian likelihood aggregation have been strong requirements on brains, and those requirements have been met by animal brains, for more than 500 million years.

## Conclusion

This article has described a promising alignment between two different approaches in the study of the brain:

1. Bayesian inference, as formalized in the Free Energy Principle, as a framework to understand active inference and scene construction as the aggregation of multiple knowledge sources.
2. The distinctive neuro-anatomy and neuro-physiology of the thalamus, whose functional anatomy is ideally suited to instantiate this aggregation.

This article speaks on an important issue: because of functional segregation in the brain, there must be an underlying architecture and computational mechanism to bring together different types of information from dynamic coalitions of knowledge sources, which have to be weighted according to their precisions—in light of (approximate) Bayesian inference. We propose that this is what the thalamus is there for. The implicit modularity of cortical representations calls upon a factorization (i.e., a mean-field approximation) that is inherent in any form of (approximate) Bayesian inference.

Active Bayesian inference and the Free Energy Principle are now firmly established as an apt explanation for many aspects of cognition. There is little doubt that they should apply to one core requirement on animal brains, which is to understand the forms and locations of objects around the animal from moment to moment, based on multi-sense-data.

For this, active inference entails a set of hypotheses about “what is where” around the animal; and various attributes (e.g., “what” and “where”) are processed by a wide range of cortical modules. The thalamus is well placed to unite these, acting as a blackboard/aggregator for hypotheses about spatial forms and positions of things in peripersonal space.

The requisite neuronal message passing and belief updating align well with many distinctive features of thalamic neuroanatomy and physiology, including:

• Quasi-independent thalamic relay cells.
• Thalamo-cortical rhythms.
• Diffuse cortical connectivity of higher-order thalamic nuclei.
• Inhibitory interneurons in the thalamus.
• The regular three-dimensional shape of the thalamus, preserved across many species.
• Olfaction bypasses the thalamus, as it does little to constrain spatial locations. Driver and modulator pathways. Triadic synapses.

Each of these features of the thalamus is consistent with a generative model that plays the role of a spatial aggregator. The alignment between the thalamus and the blackboard/aggregator model is promising at this stage. Much remains to be done to test it and validate it. If the alignment has validity, then further testing of it will involve a twin-track program:

1. Theoretical and computational investigations of the Blackboard model—to explore its viability, neural architecture requirements, scaling, and performance.
2. An empirical investigation of thalamic neuroanatomy and physiology, testing whether it is compatible with Bayesian belief updating of the sort described above.

If this is done, no doubt many of the specific proposals in this article will prove to be wrong, or need modification; but the pursuit and cross-fertilization of these twin tracks will be a productive way to increase our understanding of the thalamus (Donoho et al., 2005).

The most important proposal in this article is to suggest that the passive idea of “the thalamus as a relay” is no longer sufficient. The “relay” notion often emerges as a straightforward interpretation of experiments, but it fails to address the complexities of neuronal representation and processing not yet revealed by those experiments. As an expression of what the thalamus does, it is too weak. We should supplement the passive “relay” notion with more active notions, such as thalamic **steering** *via* precisions entailed by processed sense-data between cortical modules, and the aggregation of Bayesian beliefs—recognizing that precise spatial steering is difficult, essential, and worth doing centrally.

## Data Availability Statement

The original contributions presented in the study are included in the article, further inquiries can be directed to the corresponding author.

## Author Contributions

RW contributed ideas concerning the blackboard architecture, aggregation, steering, and thalamus training cortex. MB contributed concerning cortical knowledge sources, the alpha cycle, and amortization. VN contributed concerning the free energy principle and active inference. All authors contributed to the article and approved the submitted version.

## Conflict of Interest

MB was employed by the software company Bluecore, Inc. The remaining authors declare that the research was conducted in the absence of any commercial or financial relationships that could be construed as a potential conflict of interest.

## Footnotes

^1^In predictive coding schemes that implement free energy minimisation, the free energy gradients can be regarded as precision weighted prediction errors. More generally, these gradients reflect the discrepancy between some posterior and prior probabilistic beliefs, encoded in neuronal activity.

^2^Technically, the weight afforded different sources is determined by the precision or confidence placed in the corresponding marginal beliefs- or, when combining sense-data the precision of the likelihood of those data relative to the precision of prior beliefs about the causes of those data.

^3^This means that if two stimuli have the same or overlapping inferred locations on the blackboard, they are bound together—so that even when there are very many stimuli, the sets of stimuli which are bound together are small sets.
